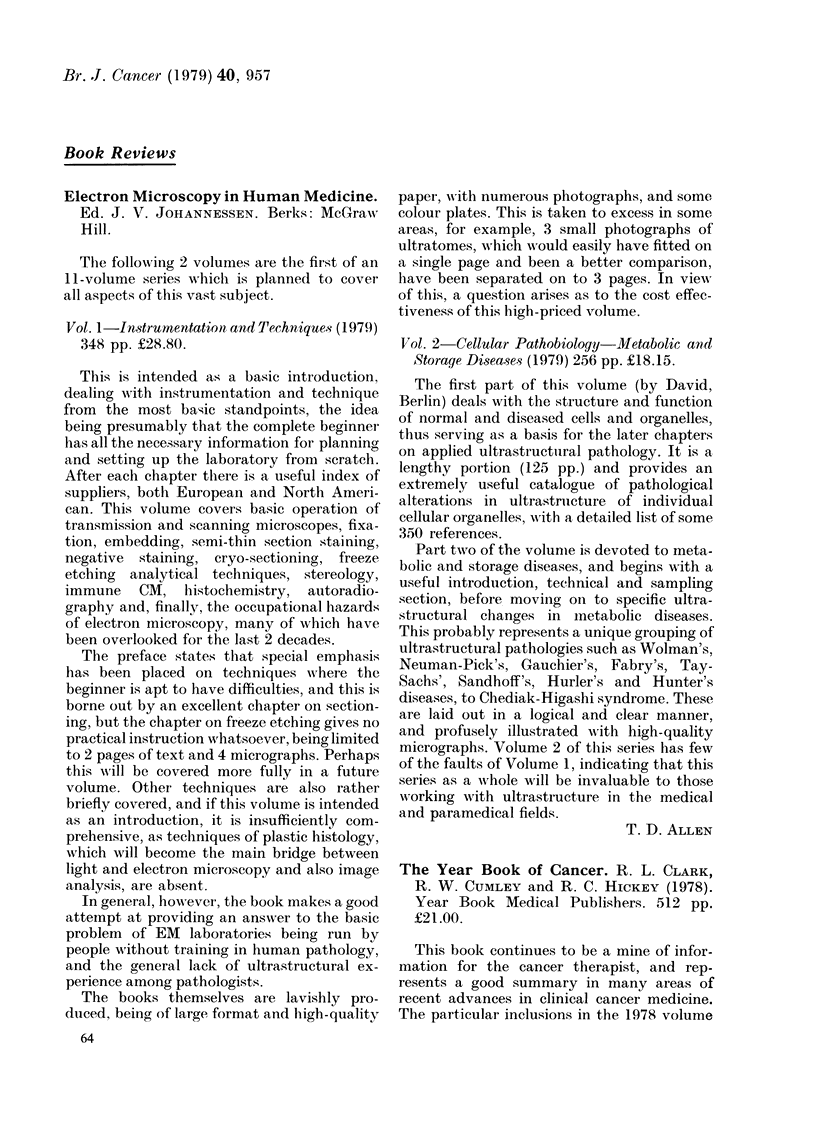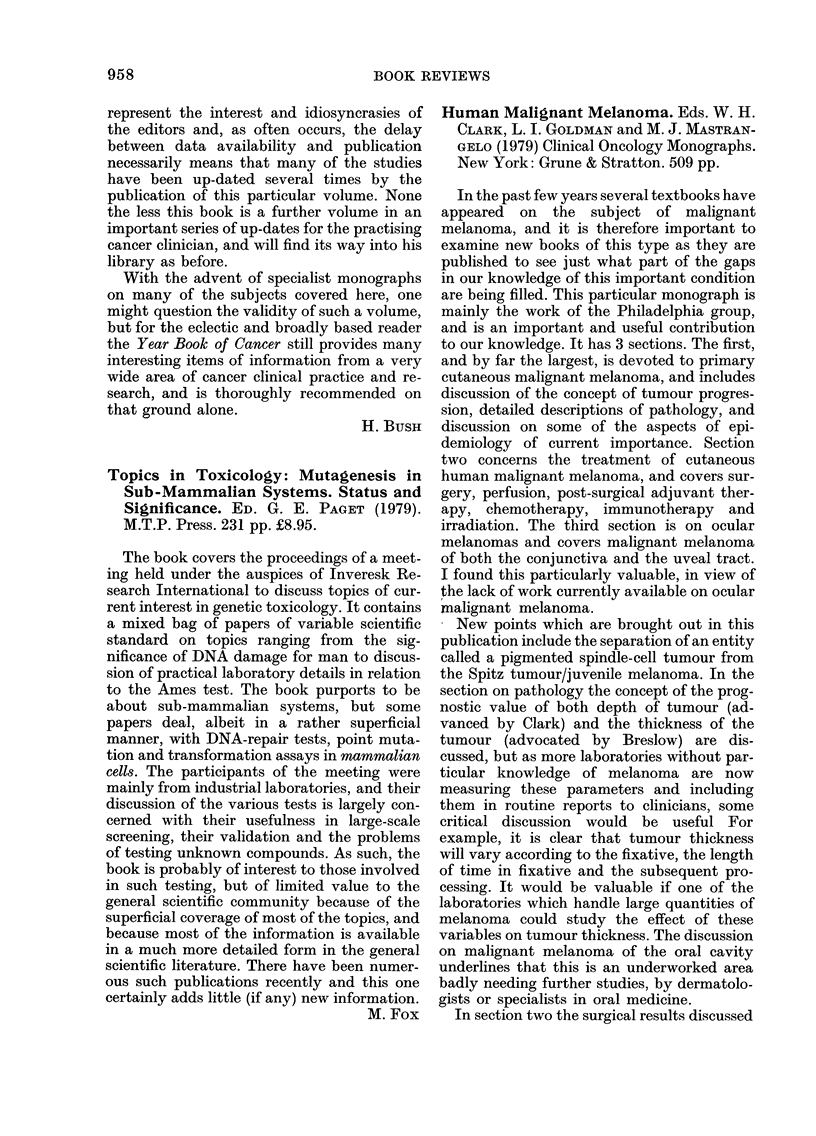# The Year Book of Cancer

**Published:** 1979-12

**Authors:** H. Bush


**The Year Book of Cancer.** R. L. CLARK, R. W. CUMLEY and R. C. HICKEY (1978). Year Book Medical Publishers. 512 pp. £21.00.

This book continues to be a mine of information for the cancer therapist, and represents a good summary in many areas of recent advances in clinical cancer medicine. The particular inclusions in the 1978 volume represent the interest and idiosyncrasies of the editors and, as often occurs, the delay between data availability and publication necessarily means that many of the studies have been up-dated several times by the publication of this particular volume. None the less this book is a further volume in an important series of up-dates for the practising cancer clinician, and will find its way into his library as before.

With the advent of specialist monographs on many of the subjects covered here, one might question the validity of such a volume, but for the eclectic and broadly based reader the *Year Book of Cancer* still provides many interesting items of information from a very wide area of cancer clinical practice and research, and is thoroughly recommended on that ground alone.

H. Bush